# Laxative effects of triple fermented barley extracts (FBe) on loperamide (LP)-induced constipation in rats

**DOI:** 10.1186/s12906-019-2557-x

**Published:** 2019-06-21

**Authors:** Jong-Min Lim, Young Dae Kim, Chang-Hyun Song, Su-Jin Park, Dong-Chan Park, Hyung-Rae Cho, Go-Woon Jung, Khawaja Muhammad Imran Bashir, Sae Kwang Ku, Jae-Suk Choi

**Affiliations:** 1 Glucan Corp, #305 Marine Bio-Industry Development Center, Hoenggye-ri 27, Ilgwang-myeon, Gijan-gun Busan, 46048 Republic of Korea; 2 South East Sea Fisheries Research Institute, National Institute of Fisheries Sciences, Tongyoung-si, Gyeongsangnam-do 53085 Republic of Korea; 3 Department of Anatomy and Histology, College of Korean Medicine, Daegu Haany University, 290 Yugok-dong, Gyeongsan-si, Gyeongsanbuk-do 38610 Republic of Korea; 4 MRC-GHF, College of Korean Medicine, Daegu Haany University, 290 Yugok-dong, Gyeongsan-si, Gyeongsanbuk-do 38610 Republic of Korea; 5 German Engineering Research and Development Center for Life Science Technologies in Medicine and Environment, 31, Gwahaksandan 1-ro, 60 beon-gil, Gangseo-gu, Busan, 46742 Republic of Korea; 6 Seafood Research Center, IACF, Silla University, 606, Advanced Seafood Processing Complex, Wonyang-ro, Amnam-dong, Seo-gu Busan, 49277 Republic of Korea; 7 Division of Bioindustry, College of Medical and Life Sciences, Silla University, 140 Baegyang-daero, 700 beon-gil, Sasang-gu Busan, 46958 Republic of Korea

**Keywords:** Crl:CD [SD] rats, Laxative effects, Loperamide-induced constipation, Triple fermented barley extract

## Abstract

**Background:**

Constipation, a common health problem, causes discomfort and affects the quality of life. This study intended to evaluate the potential laxative effect of triple fermented barley (*Hordeum vulgare* L.) extract (FBe), produced by saccharification, *Saccharomyces cerevisiae,* and *Weissella cibaria*, on loperamide (LP)-induced constipation in Sprague-Dawley (SD) rats, a well-established animal model of spastic constipation.

**Methods:**

Spastic constipation was induced via oral treatment with LP (3 mg/kg) for 6 days 1 h before the administration of each test compound. Similarly, FBe (100, 200 and 300 mg/kg) was orally administered to rats once a day for 6 days. The changes in number, weight, and water content of fecal, motility ratio, colonic mucosa histology, and fecal mucous contents were recorded. The laxative properties of FBe were compared with those of a cathartic stimulant, sodium picosulfate. A total of 48 (8 rats in 6 groups) healthy male rats were selected and following 10 days of acclimatization. Fecal pellets were collected one day before administration of the first dose and starting from immediately after the fourth administration for a duration of 24 h. Charcoal transfer was conducted after the sixth and final administration of the test compounds.

**Results:**

In the present study, oral administration of 100–300 mg/kg of FBe exhibited promising laxative properties including intestinal charcoal transit ratio, thicknesses and mucous producing goblet cells of colonic mucosa with decreases of fecal pellet numbers and mean diameters remained in the lumen of colon, mediated by increases in gastrointestinal motility.

**Conclusion:**

Therefore, FBe might act as a promising laxative agent and functional food ingredient to cure spastic constipation, with less toxicity observed at a dose of 100 mg/kg.

## Background

Constipation is a widespread functional gastrointestinal illness that affects 3–15% of general population and causes discomfort and negative impact on quality of life [1–4]. It can also cause restlessness, vomiting, gut obstruction, perforation, and may be linked with fatal pulmonary embolism or aspiration [5]. Currently, 20–30% of people older than 60 years use more than one laxative per week [6]. Drugs containing sennoside or magnesium oxide have powerful laxative/purgative activity and are mainly prescribed for constipation related illnesses; however, these drugs also induce side effects such as severe diarrhea [2], and their frequent use can induce melanosis coli, a risk factor for colorectal neoplasm [7].

There has recently been a rise in attention towards the role of functional foods in maintaining well-being, resulting in an increased demand for functional foods produced from natural sources [8]. Natural products are gaining interest in the biopharmaceutical industry as well as inspiring the search for novel potential sources of bioactive metabolites [9, 10]. Medicinal plants, crude drug substances as well as several herbs have antioxidant properties [11]. Grain consumption has been enhanced due to their favorable effects with respect to lowering the risk of diabetes, cardiovascular diseases, ischemic stroke, metabolic syndromes, and gastrointestinal cancer [12–14]. Grains contain minerals, vitamins, phytochemicals and functional dietary fibers that are favorable for human body [14, 15]. Recently, fermented herbs have also been proposed as a potential source of medicinal and pharmaceutical ingredient, particularly since fermentation is believed to enhance the bioactivity of natural herbs through probiotic effect and biotransformation [16–23].

Globally, barley grain is used in the brewing industry as a non-toxic cereal grain [24]. Furthermore, it is also used as an ingredient in various foods, beverages and animal forage [24]. The phenolic compounds present in barley (*Hordeum vulgare* L.) have shown antioxidants effects in the promotion of health [14, 25, 26]. This includes anticancer [24] and probiotic gastroprotective effects [27]. The functionality and bioavailability of these phenolic compounds is increased by fermentation process [28, 29], particularly the antioxidative effect [14, 15]. Various fermented barley extracts (FBe) have shown a number of potent pharmacological effects, especially improved antioxidative [14], uric acid-lowering [30], anti-atopic dermatitis [31, 32], hepatoprotective [14, 15], and immunostimulatory effects [33], compared with non-fermented extracts.

Rats have typically been used as experimental animals to test the efficacy of various drugs. Dietary habits, chemical compounds such as morphine, and psychological stress have been considered as the causes of constipation [2, 20–22]. Normal rats are also useful experimental animals with regard to detecting various digestive disorders [20, 34–36]. It has been established that loperamide (LP) can induce delays in colonic transit due to its inhibition of stool frequency in mice and increase in colonic contractions, resulting in spastic constipation [37]. This drug has been shown to inhibit colonic peristalsis and intestinal water secretion [38, 39], ultimately, extending the fecal evacuation time and delaying the intestinal luminal transit [40]. Therefore, LP-induced constipation has been considered a suitable animal model of spastic constipation [21, 22, 41].

The laxative effects of triple fermented rice extract by saccharification, *Saccharomyces*, and *Weissella* in normal rats [21] and in loperamide-treated rats [22] have been reported. Previously, we reported the less toxic behavior of the triple fermented barley extract using saccharification [20–23]. However, there are currently no systematic assessments of the laxative effects of FBe in rodent models. Therefore, this study was intended to test the potential enhanced laxative effects of FBe in rat models of LP-induced constipation, using methods established in our previous studies [21, 22].

## Materials and methods

### Experimental animals

**Fig. 1:**
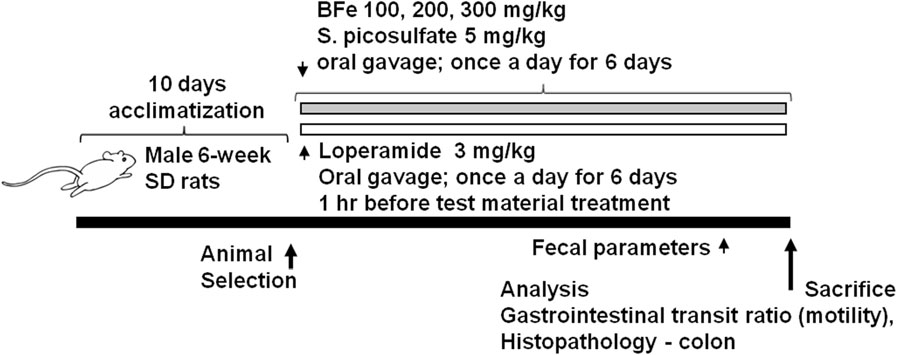
Experimental designs used in this study. FBe: Triple fermented barley extracts, test material; LP: Loperamide; SP: Sodium picosulfate

A total of 60 healthy 6-week old male SPF/VAF Outbred Crl:CD [SD] rats were purchased from OrientBio, Seungnam, Republic of Korea and acclimatized for 10 days prior to use for the experiments. Animal husbandry conditions were similar to our previously reported studies [20, 21]. A total of 48 rats (8 rats in 6 groups) were selected on body weight basis (mean: 262.17 ± 12.84 g, range: 239.00~288.00 g) and fecal water content basis (mean: 31.36 ± 5.83%, range: 20.23~41.33%) measured one day before administration of the first dose of the test material. The rats in the 6 experimental groups were sacrificed and analyzed (Table 1 and Fig. 1).

**Table 1:** Composition of barley extract (Be) and fermented barley extract (FBe)

Nutrition Fact	Unit	Amount	Amount
		Be	FBe
Calorie	kcal/100 g	361.3	385.3
Carbohydrates	%	79.0	93.0
Protein	%	9.4	3.1
Lipids	%	1.1	0.1
Total polyphenols	mg/g	0.493 ± 0.102	3.66 ± 0.12
Total Flavonoids	mg/g	0.159 ± 0.068	0.31 ± 0.02
Dietary fiber	%	12.10	20.20

### Experimental sample preparation and administration

The FBe used in this study were prepared as demonstrated in our previous reports [20–23]. This final fermentate was steam sterilized (VS-1321-80; Vision Scientific Co. Ltd., Daejeon, Korea) and filtered through a 40 mesh sieve to obtain the final filtrate. The positive control sodium picosulfate (SP) was used as a reference drug as suggested by the previously reported studies [42, 43]. Some FBe specimens [Code FBe2014Ku01] were deposited in the herbarium of the Medical Research Center for Globalization of Herbal Formulation, Daegu Haany University, Republic of Korea. FBe was dissolved in distilled water to a final concentration of 100, 200, and 300 mg/kg and orally administered once a day for 6 days (in a volume of 5 ml/kg) 1 h after each LP administration as suggested by the previously reported studies [21, 22]. SP, dissolved in distilled water, was also orally administered at a dose of 5 mg/kg [21, 22, 44]. For intact LP control rats, distilled water (5 ml/kg) was administered once a day for 6 days via gastric gavage, in place of the test solutions.

### Composition analysis of FBe

Compositional analyses were performed according to the reported methods of Food Code [45] and Health Functional Food Code [46].

### Induction of constipation in the rats

Constipation was induced in the animals through oral administration of 3 mg/kg of LP, once a day for 6 continuous days at 1 h before administration of each test material [21, 22, 47, 48]. The intact control rats were administered saline only.

### Changes in body weight

The daily body weights of individual rats were measured starting from one day before administration of the test compounds through the sixth day of administration of the test compounds and LP. All rats were fasted overnight (water was provided; approximately 12–18 h) before the first administration and at termination to reduce variations in weight associated with feeding and for intestinal charcoal transfer measurement. Furthermore, body weight gains during the administration period were calculated using Eq. 1 as follows.

1 $$ \mathrm{Body}\ \mathrm{weight}\ \mathrm{gain}\ \left(\mathrm{g}\right)=\mathrm{body}\ \mathrm{weight}\ \mathrm{on}\ \mathrm{sixth}\ \mathrm{day}\ \mathrm{of}\ \mathrm{administration}-\mathrm{body}\ \mathrm{weight}\ \mathrm{before}\ \mathrm{first}\ \mathrm{administration} $$

### Measurement of fecal parameters

The excreted fecal pellets of individual rats during a 24-h period were collected one day before first administration of the test compound and immediately after the fourth administration for a duration of 24 h. The total number, water content and wet-weight of the fecal pellets were calculated. The collected fecal pellets were dried at 60 °C in a general dry oven for 24 h to obtain the fecal dry weights. The water content was calculated using Eq. 2.

2 $$ \mathrm{Fecal}\ \mathrm{pellet}\ \mathrm{water}\ \mathrm{contents}\ \left(\%\right)=\left[\left(\mathrm{fecal}\ \mathrm{wet}-\mathrm{weigh}\mathrm{t}-\mathrm{fecal}\ \mathrm{dry}-\mathrm{weigh}\right)/\mathrm{fecal}\ \mathrm{wet}-\mathrm{weigh}\mathrm{t}\right]\times 100 $$

### Measurement of intestinal charcoal transit ratio

Gastrointestinal propulsion of a charcoal meal was measured according as described by Sagar et al. [49] with slight modifications. Test animals were fasted 18 h prior to the experiment. Ten minutes after the last dose of the test compound (sixth day of administration), the animals from each group were fed 1 ml of a charcoal meal containing 3% suspension of activated charcoal in 0.5% aqueous methylcellulose (Sigma-Aldrich Co. Ltd., St. Louise, MO, USA). Thirty minutes after administration of the charcoal meal, 99.0% CO_2_ gas as a euthanasia agent was used, for euthanasia of rodents [50] and the animals were sacrificed via cervical dislocation. The intestinal charcoal transit ratio was estimated following the Eq. 3.

3 $$ \mathrm{Charcoal}\ \mathrm{transit}\ \mathrm{ratio}\ \left(\%\right)=\left[\left(\mathrm{total}\ \mathrm{small}\ \mathrm{intestine}\ \mathrm{length}-\mathrm{charcoal}\ \mathrm{meal}\ \mathrm{transfer}\ \mathrm{length}\right)/\mathrm{total}\ \mathrm{small}\ \mathrm{intestine}\ \mathrm{length}\right]\times 100 $$

### Measurement of fecal pellets in large intestine

After the measurement of intestinal charcoal transit ratios, the total number and mean thickness (short axis) of fecal pellet remnants in the colon lumen were quantified individually.

### Histological procedures

Histological observations of colon mucosa and fecal pellet remnants in the colon lumen were performed according to the previously reported method of Wu et al. [51] with slight modifications. The segments of the rat distal colon were fixed with 10% neutral buffered formalin (NBF), embedded in paraffin, serially cut into 3 μm think cross sections, and stained with alcian blue (pH = 2.5). To observe detailed changes in the mean thickness of the mucosal layers at the fecal surface, the number of mucous-producing cells and colonic mucosa thickness were measured as part of the histomorphometry assessment using iSolution FL ver 9.1 (a computer-based image analyzer; IMT i-solution Inc., Quebec, Canada) under Eclipse 80i microscope (Nikon, Tokyo, Japan). The samples were randomly number to limit the possible biasness caused by the histopathologist during the analyses.

### Statistical analyses

Different dose groups were compared by multiple comparison tests. Levene’s test was performed to examine variance homogeneity [52]. In case of no significant deviation observed by Levene’s test, the data were analyzed using one-way analysis of variance (ANOVA) tests followed by least-significant differences (LSD) multi-comparison tests. If a significant deviation from variance homogeneity was observed, a non-parametric comparison test, the Kruskal-Wallis H test, was performed. In case of significant differences in the Kruskal-Wallis H test, the Mann-Whitney U (MW) test was performed to examine the significantly different pairs [53]. Results were considered significant at *p* < 0.05. Statistical analyses were performed on SPSS ver. 14 (SPSS Inc., Chicago, IL, USA). Furthermore, the percentage point changes between intact vehicle and LP control were measured to observe the severity of spastic constipation induced by LP treatment (Eq. 4). The percentage point difference between LP control and treated rats were also examined to further examine the laxative effects of the test compounds (Eq. 5). These quantifications were performed according to the previously reported method [54].

4 $$ \mathrm{Percentage}\ \mathrm{point}\ \mathrm{change}\ \mathrm{compared}\ \mathrm{with}\ \mathrm{intact}\ \mathrm{vehicle}\ \mathrm{control}\ \left(\%\right)=\left[\left(\mathrm{LP}\ \mathrm{control}\ \mathrm{rats}-\mathrm{intact}\ \mathrm{vehicle}\ \mathrm{control}\ \mathrm{rats}\right)/\mathrm{intact}\ \mathrm{vehicle}\ \mathrm{control}\ \mathrm{rats}\right]\times 100 $$

5 $$ \mathrm{Percentage}\ \mathrm{point}\ \mathrm{change}\ \mathrm{compared}\ \mathrm{with}\ \mathrm{LP}\ \mathrm{control}\ \left(\%\right)=\left[\left(\mathrm{test}\ \mathrm{compound}\ \mathrm{treated}\ \mathrm{rats}-\mathrm{LP}\ \mathrm{control}\ \mathrm{rats}\right)/\mathrm{LP}\ \mathrm{control}\ \mathrm{rats}\right]\times 100 $$

## Results

### Nutritional composition of FBe

Compositional analyses showed a nutritional composition of calories, proteins, carbohydrates, dietary fiber and lipids FBe as 385.3 kcal 100 g^− 1^, 3.1, 93.0, 20.20, and 0.1%, respectively. Total flavonoids and total polyphenol contents were 0.31 mg g^− 1^ and 3.66 mg g^− 1^, respectively (Table 1). An increase in dietary fiber content and total polyphenols was observed, probably due to fermentation.

### Effect on body weight and weight gain

**Fig. 2:**
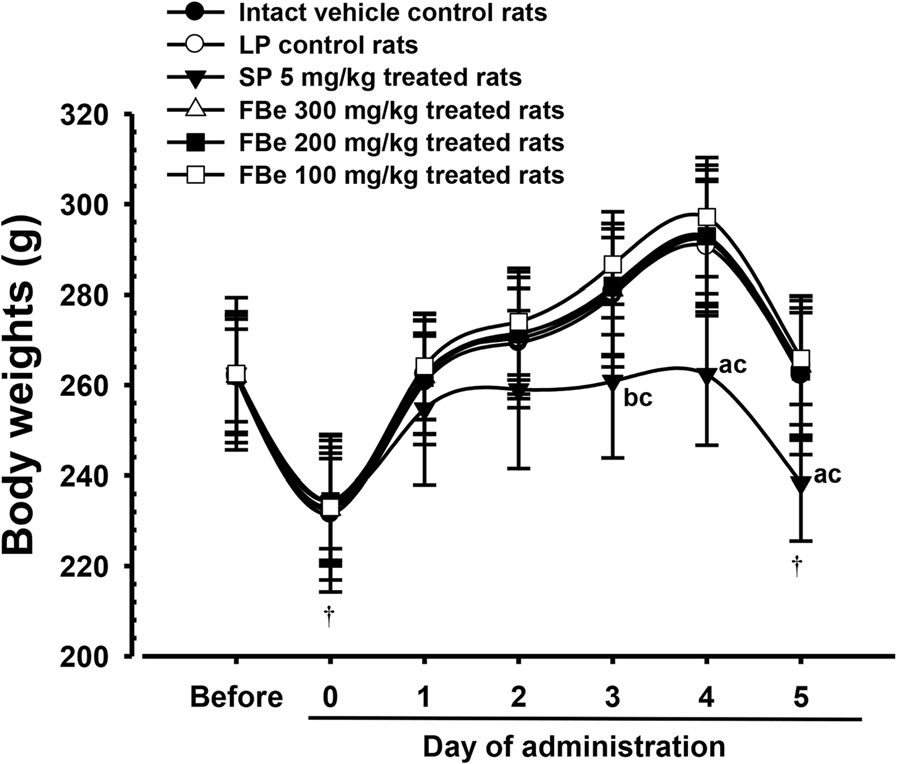
Body weight changes in the constipation rats induced by LP. Values are expressed mean ± SD of eight rats, g. FBe: Triple fermented barley extracts, test material; SP: Sodium picosulfate; LP: Loperamid. All animals were overnight fasted before fist and last sixth test material administration (**†**). Before means 1 day initiation of administration and 0 means at initiation of administration. ^a^ *p* < 0.01 and ^b^ *p* < 0.05 as compared with intact control by LSD test. ^c^ *p* < 0.01 as compared with LP control by LSD test

Significant decreases in body weights 3 days after first administration (*p* < 0.01; *p* < 0.05) as well in body weight gains from first to final administration (*p* < 0.01) were noted in SP-treated rats compared with intact and LP control rats. However, compared to intact vehicle controls, there were no LP treatment-related changes in body weight gains. Additionally, none of the treated groups (100–300 mg/kg FBe) showed a meaningful change in body weight and weight gain as compared with LP control rats (Table 2; Fig. 2).

**Table 2:** Body weight gains in the constipation rats induced by LP

Time/Index Groups	Body weights at			Body weight gains [B-A]
	1 day before first treatment	First treatment day* [A]	Last (6th) treatment day* [B]	
Control				
Intact	261.75 ± 14.51	231.50 ± 17.25	262.13 ± 17.52	30.63 ± 4.21
LP	262.25 ± 13.23	233.13 ± 13.08	263.25 ± 15.48	30.13 ± 7.49
Reference				
SP 5 mg/kg	262.50 ± 12.95	234.25 ± 13.50	238.38 ± 12.82^ab^	4.13 ± 3.83^ab^
FBe treated as				
300 mg/kg	261.88 ± 12.77	232.50 ± 11.20	264.13 ± 15.64	31.63 ± 6.74
200 mg/kg	262.13 ± 10.26	234.38 ± 10.51	263.00 ± 14.11	28.63 ± 9.01
100 mg/kg	262.50 ± 16.86	233.00 ± 16.07	265.88 ± 10.19	32.88 ± 6.58

Values are expressed mean ± S.D. of eight rats, g

FBe = Triple fermented barley extracts, test material

SP = Sodium picosulfate

LP = Loperamide

*Overnight fasted

^a^*p* < 0.01 as compared with intact control by LSD test

^b^*p* < 0.01 as compared with LP control by LSD test

### Changes in fecal parameters

Fecal wet-weight, fecal dry-weight, fecal number, and water content were similar in all rats co-administered LP with/without the test substance at baseline (collected for 24 h) compared to intact vehicle control rats. However, significant decreases in fecal number and water content were detected following the fifth treatment day in LP controls compared to intact vehicle controls (*p* < 0.01). Dose-dependent significant increase in water content and fecal number was also demonstrated for the three different dosages of FBe (100–300 mg/kg) compared with the LP control, based on data collected for 24 h starting from immediately after the fourth administration (*p* < 0.01 or *p* < 0.05). Furthermore, administration of 5 mg/kg SP in rats showed a significant increase in water content and fecal number compared to LP control rats (*p* < 0.01; data collected prior to the fifth administration; Table 3).

**Table 3:** Fecal parameters in the constipation rats induced by LP

Time/Index Groups	Collected at 1 day before treatment for 24 h				Collected from after 4th administration for 24 h			
	Pellet numbers = (numbers/rat)	Wet-weights (g/rat)	Dry-weights (g/rat)	Water contents (%/rat)	Pellet numbers (numbers/rat)	Wet-weights (g/rat)	Dry-weights (g/rat)	Water contents (%/rat)
Control								
Intact	60.25 ± 12.43	9.72 ± 1.46	6.67 ± 1.57	32.06 ± 3.76	57.75 ± 11.67	9.71 ± 1.29	6.63 ± 1.23	32.16 ± 3.69
LP	61.25 ± 12.52	9.65 ± 0.91	9.74 ± 0.92	30.33 ± 3.24	32.50 ± 6.21^a^	3.81 ± 0.75^a^	3.34 ± 0.58^a^	11.91 ± 4.04^a^
Reference								
SP 5 mg/kg	62.50 ± 13.21	10.37 ± 1.44	7.39 ± 1.55	29.38 ± 5.97	67.88 ± 11.34^c^	10.66 ± 1.35^c^	7.14 ± 1.10^c^	33.08 ± 5.68^c^
FBe treated as								
300 mg/kg	62.25 ± 11.18	9.61 ± 1.45	6.48 ± 1.48	33.10 ± 5.62	59.00 ± 11.64^c^	9.73 ± 1.37^c^	7.11 ± 1.05^c^	26.99 ± 2.71^bc^
200 mg/kg	60.75 ± 16.59	9.96 ± 1.73	6.87 ± 1.84	31.90 ± 6.26	54.38 ± 10.93^c^	9.28 ± 1.30^c^	7.08 ± 0.94^c^	23.51 ± 5.18^ac^
100 mg/kg	60.88 ± 13.65	10.10 ± 1.32	7.01 ± 1.66	31.43 ± 7.86	44.00 ± 7.69^bd^	8.21 ± 0.79^bc^	6.73 ± 0.57^c^	18.01 ± 2.77^ac^

Values are expressed mean ± S.D. of eight rats

FBe = Triple fermented barley extracts, test material

SP = Sodium picosulfate

LP = Loperamide

To determine fecal dry weights, all collected fecal pellets were dried at 60 °C in a general dry oven for 24 h

Fecal pellet water contents (%) = [(fecal wet-weight – fecal dry-weigh)/fecal wet-weight] × 100

^a^*p* < 0.01 and ^b^ *p* < 0.05 as compared with intact control by LSD test

^c^*p* < 0.01 and ^d^ *p* < 0.05 as compared with LP control by LSD test

### Effect on remnant fecal pellets in the colon lumen

**Fig. 3:**
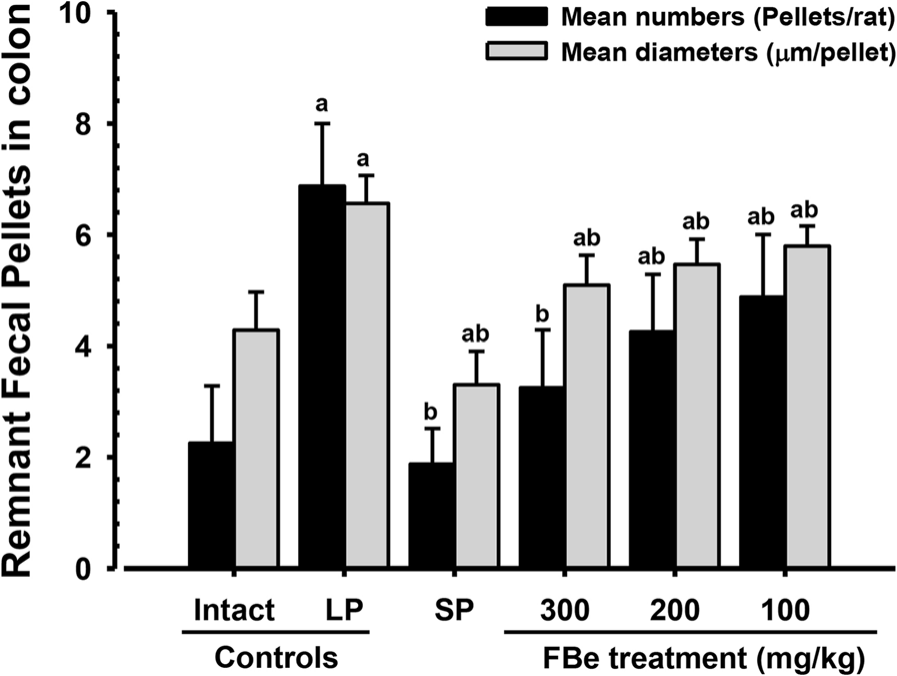
Fecal pellets remnant in the lumen of colon of the constipation rats induced by LP. Values are expressed mean ± SD of eight rats. FBe: Triple fermented barley extracts, test material; SP: Sodium picosulfate; LP: Loperamide. ^a^ *p* < 0.01 as compared with intact control by LSD test. ^b^ *p* < 0.01 as compared with LP control by LSD test

LP control rats showed significant increases in remnant fecal numbers in the colon lumen and the corresponding mean diameters at sacrifice compared with intact vehicle control rats following the 18-h fast (*p* < 0.01). However, at sacrifice, significant decreases in remnant fecal number in the colon lumen and the corresponding mean diameters were observed in rats treated with 5 mg/kg SP and all dosages of FBe compared with vehicle control rats (*p* < 0.01). Furthermore, FBe-treated rats showed clear dose-dependent decreases in remnant fecal number and mean diameters in colon lumen (Fig. 3).

### Effect on intestinal charcoal transit

Significant decreases in intestinal charcoal transit ratio were observed in LP control rats compared with vehicle control rats (*p* < 0.01). However, compared with LP, significant dose-dependent increase in intestinal charcoal transit ratio was observed following 6 days of continuous oral co-treatment with three different dosages of FBe (*p* < 0.01; *p* < 0.05). In addition, the groups treated with 5 mg/kg SP revealed a significant increase in intestinal charcoal transit ratio compared with the LP control group (*p* < 0.01; Table 4).

**Table 4:** Gastrointestinal charcoal transit ratio in the constipation rats induced by LP

Groups	Gastrointestinal motilities (during 30 min)		
	Total small intestine length (cm)	Length of charcoal meal transferred (cm)	Gastrointestinal charcoal transit ratio (%)
Control			
Intact	107.53 ± 7.38	77.45 ± 5.87	72.07 ± 3.52
LP	106.60 ± 8.54	42.93 ± 10.89^a^	39.65 ± 10.50^a^
Reference			
SP 5 mg/kg	108.71 ± 9.61	57.10 ± 11.31^ac^	52.57 ± 9.58^ac^
FBe treated as			
300 mg/kg	107.99 ± 8.74	67.44 ± 10.91^bc^	63.19 ± 13.10^c^
200 mg/kg	109.14 ± 9.88	60.98 ± 10.63^ac^	55.84 ± 8.18^ac^
100 mg/kg	108.33 ± 6.11	55.90 ± 4.68^ac^	51.64 ± 3.91^ad^

Values are expressed mean ± S.D. of eight rats

FBe = Triple fermented barley extracts, test material

SP = Sodium picosulfate

LP = Loperamide

Charcoal transit ratio (%) = [(Total small intestine length – Length of charcoal meal transferred)/Total small intestine length] × 100

^a^*p* < 0.01 and ^b^ *p* < 0.05 as compared with intact control by LSD test

^c^*p* < 0.01 and ^d^ *p* < 0.05 as compared with LP control by LSD test

### Histopathological analysis

**Fig. 4:**
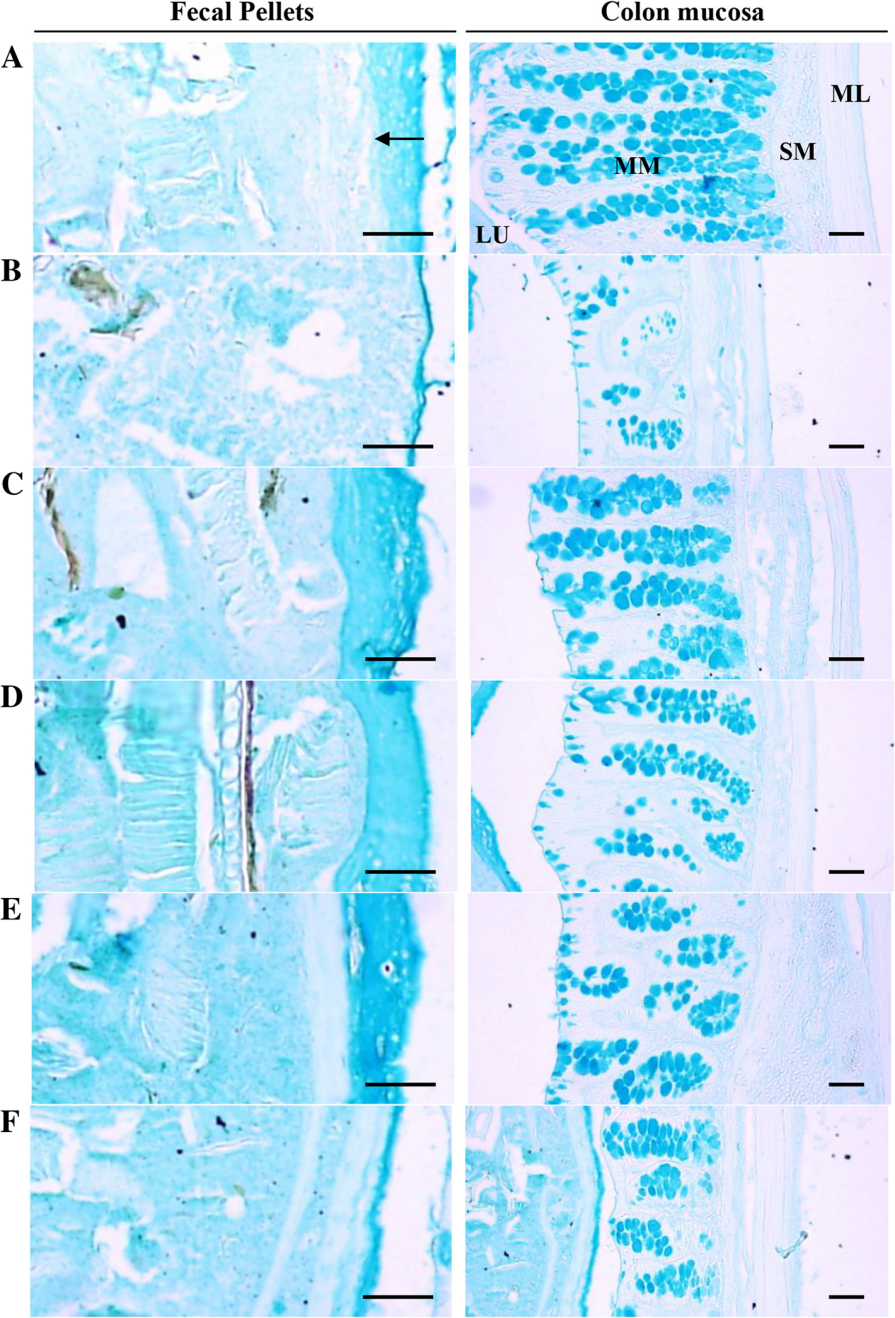
Representative histological images of the colon contains fecal pellet, taken from vehicle, SP or FBe-treated normal rats. **a** = Intact vehicle control (Saline and distilled water administered intact vehicle control rats). **b** = LP control (LP 3 mg/kg and distilled water administrated constipation control rats). **c** = Reference (LP 3 mg/kg and SP 5 mg/kg administered rats). **d** = FBe 300 (LP 3 mg/kg and FBe the highest dosage 300 mg/kg administered rats). **e** = FBe 200 (LP 3 mg/kg and FBe the middle dosage 200 mg/kg administered rats). **f** = FBe 100 (LP 3 mg/kg and FBe the lowest dosage 100 mg/kg administered rats). FBe: Triple fermented barley extracts, test material; MM: Colonic mucosa; LU: Lumen; SM: Submucosa: ML: Muscular layer. Arrow indicated surface mucous thicknesses of fecal pellets measured. All alcian blue stain Scale bars = 120 μm

Significant decreases in the number of mucous-producing cells in the colonic mucosa, the surface mucous thickness of colon lumen remnant fecal pellets (at sacrifice) and the mean colonic mucosa thickness following 6 days of continuous oral treatment with LP (3 mg/kg) compared with vehicle control (*p* < 0.01) were observed. However, significant increases in the number of mucous-producing cells and the surface mucous thickness of colon lumen remnant fecal pellets were observed after 6 days of continuous oral co-treatment of SP 5 mg/kg (*p* < 0.01). A dose-dependent increase in these parameters for rats treated with FBe (100–300 mg/kg) was also observed. In addition, the colonic mucosa thickness also significantly increased in rats treated with SP 5 mg/kg, as well as dose-dependently increased in rats treated with FBe (100–300 mg/kg), compared with that in vehicle control rats (*p* < 0.01; Table 5; Fig. 4).

**Table 5:** Histomorphometrical analysis of the colon and remnant fecal pellets in the constipation rats induced by LP

Groups	Histomorphometry (at sacrifice)		
	Fecal pellet surface mucous thicknesses (μm)	Mucous producing cell numbers (cells/mm^2^)	Colon mucosa thicknesses (μm)
Control			
Intact	62.28 ± 17.39	610.38 ± 140.52	523.25 ± 118.89
LP	14.42 ± 2.05^a^	148.50 ± 46.34^a^	214.56 ± 38.30^a^
Reference			
SP 5 mg/kg	101.83 ± 17.48^ac^	482.25 ± 57.62^c^	405.61 ± 51.15^bc^
FBe treated as			
300 mg/kg	76.09 ± 13.22^c^	380.25 ± 70.14^ac^	362.15 ± 56.27^ac^
200 mg/kg	54.12 ± 12.84^c^	308.13 ± 45.17^ac^	314.98 ± 86.75^ac^
100 mg/kg	26.68 ± 9.42^ac^	274.88 ± 60.44^ac^	273.14 ± 22.32^ac^

Values are expressed mean ± S.D. of eight rat samples

FBe = Triple fermented barley extracts, test material

SP = Sodium picosulfate

LP = Loperamide

^a^ *p* < 0.01 and ^b^ *p* < 0.05 as compared with intact control by MW test

^c^ *p* < 0.01 as compared with LP control by MW test

## Discussion

Constipation can arise from a variety of sources, including dietary habits, chemical compounds such as morphine and psychological stress [2]. It increases with age and may necessitate long-term treatment with laxatives. In the present study, in order to evaluate the potential laxative effects of FBe, we examined changes in fecal parameters (i.e., weight, numbers, and water content), fecal mucous content, gastrointestinal transit ratio (motility), and colonic mucosa histology (i.e., mean colonic mucosa thickness under alcian blue stain, number of colonic mucous-producing cells, and mean mucous membrane thickness of fecal pellets in the colon lumen) in LP-induced rats, a suitable animal model of spastic constipation [21, 22, 41]. The laxative effects of FBe were compared with SP (5 mg/kg), a cathartic stimulant activated by colonic bacteria [42, 43], as the reference drug [21, 22, 46].

Spastic constipation was induced via oral treatment with LP (3 mg/kg) once a day for 6 consecutive days 1 h before test substance administration, in accordance with our previous studies [21, 22, 47, 48]. The dosages of FBe (100–300 mg/kg) were selected according to our previous studies of fermented rice extracts in LP-induced constipation rat models [21, 22]. The 5 mg/kg dose of SP was also selected according to the previous studies [21, 22, 44]. Fecal pellets were collected one day before the first dose of the test substance and starting from immediately following the fourth administration for a 24-h duration, in order to measure fecal parameters and select the appropriate animals. Charcoal transfer was conducted after the sixth administration of the test substances.

A continuous oral supply of LP (3 mg/kg) for 6 days showed significant decreases in fetal water content and fecal pellet number, intestinal charcoal transit ratio, surface mucous thickness of fecal pellets found in the colon lumen at sacrifice, number and thickness of mucous-producing goblet cells in the colonic mucosa, and the mean diameter and number of fecal pellets remaining in the colon lumen at sacrifice. These findings are consistent with the classic signs of LP-induced spastic constipation [21, 22, 41]. However, these LP-induced spastic constipation-related decreases in intestinal motility and fecal discharge, as well as the histopathological changes in fecal and colon pellets in the colon lumen were significantly and dose-dependently inhibited by additional continuous oral administration of FBe (100–300 mg/kg) for 6 days. These findings provide evidence for the laxative effect of FBe on LP-induced spastic constipation in rats, without causing excess diarrhea. Thus, FBe at 100 mg/kg doses may act as a potent functional food ingredient or laxative agent to treat spastic constipation with low toxicity [55]. Our results also showed that the laxative effects of FBe (300 mg/kg) were milder than those of SP (5 mg/kg). However, favorable increases in intestinal motility and charcoal transit ratio were demonstrated with FBe 300 mg/kg and 200 mg/kg than with SP (5 mg/kg). FBe 100 mg/kg also showed similar inhibitory effects on the LP-induced decreased intestinal motility as SP 5 mg/kg.

No LP-related changes in body weight and weight gains were observed compared to intact vehicle control, which was similar to the results of our previous studies [21, 22]. Additionally, no meaningful changes in body weight and weight gains were detected for three different dosages of FBe (100, 200, and 300 mg/kg) compared with LP. It should be noted that FBe did not induce severe diarrhea as a side effect since FBe showed milder and favorable laxative effects compared with (SP 5 mg/kg). Furthermore, FBe did not impact body weight and weight gains. Contrastingly, SP (5 mg/kg) induced significant decreases in body weight and weight gains compared with both LP and vehicle controls, possibly due to its long-established powerful purgative and laxative activity [42, 43]. All rats used in this study in the intact control, LP control, and all FBe-treated groups showed body weight increases that were within the range for normal age-matched rats [56, 57].

Marked decreases in fecal discharge are typically seen in constipation; specifically, the delay of fecal pellets in the large intestinal lumen can induce over-absorption of water and subsequently, the water content of the discharged pellets are significantly decreased. Therefore, fecal parameters such as number of fecal water content and fecal pellets discharged are valuable indices for determining the effects of various laxative agents [44, 48]. LP has been shown to induce noticeable decreases in fetal water content and fecal pellet number as indications of spastic constipation [21, 22]. The increased fecal water content and fecal pellet number discharged detected in rats treated with FBe (100–300 mg/kg) compared with LP control rats suggest that FBe has promising laxative properties on spastic constipation. Decreases in fecal surface mucous content and increases in remnant fecal pellet number in the colon lumen have previously been observed in constipation [20, 36, 46, 58] as well as treatment with LP [21, 22]. Our results showed decreases in remnant fecal pellet number in the colon lumen and increases in surface mucous content following treatment with FBe (100–300 mg/kg), providing support for the hypothesis that FBe has promising laxative effects at these doses. In this study, rats treated with SP (5 mg/kg) also showed significant increases in the fecal water content, number of fecal pellets discharged, fecal water content, and the surface mucous thickness of remnant pellets in the colon lumen.

LP has been shown to decrease gastrointestinal charcoal transit ratio, a marker of intestinal motility, consistent with signs of spastic constipation [21, 22]. These signs were also observed in the LP-treated control rats used in the present study. Therefore, significant and dose-dependent increases in gastrointestinal charcoal transit ratio in rats treated with FBe (100–300 mg/kg) compared to LP control provide indirect evidence that FBe has promising laxative effects against LP-induced spastic constipation. Significant increases in intestinal motility, as measured by charcoal transit ratio, were demonstrated with FBe 300 mg/kg and 200 mg/kg compared with SP (5 mg/kg), and rats treated with FBe 100 mg/kg exhibited similar inhibitory effects on the LP-induced decreases in intestinal motility compared with SP 5 mg/kg.

Reduction in mucous production in the colonic mucosa on histopathological assessments are directly related with constipation [58]; specifically, marked decreases in the thickness of the colonic mucosa layer and mucous-producing cells have been observed [20–22, 36, 59]. Additionally, treatment with 3 mg/kg of LP has been associated with marked decreases in mucosa thickness and colonic mucous-producing cells [21, 22]. In the present study, compared with intact controls, significant decreases in the surface mucous thickness of remnant fecal pellets found in the colon lumen at sacrifice, the number of mucous-producing cells in the colonic mucosa, and the mean colonic mucosa thickness were detected in rats following 6 days of consecutive oral application of LP (3 mg/kg). However, compared with LP controls, co-treatment with SP (5 mg/kg) and FBe (100–300 mg/kg) was associated with a significant increase in the number of mucous-producing cells in the colonic mucosa and the surface mucous thickness of remnant fecal pellets in the colon lumen. The effects of FBe were found to be dose-dependent. In addition, the colonic mucosa thickness significantly increased in rats treated with SP (5 mg/kg) and FBe (100–300 mg/kg; dose-dependent) compared with that in vehicle control rats.

Total polyphenols, total flavonoids and dietary fiber content of FBe were 3.66, 0.31 and 20.20%, respectively (Table 1). According to the meta-analysis of Yang et al. [60], intake of dietary fiber can clearly increase stool frequency in patients with constipation. Thus, a possible mechanism with which FBe improved the constipation seems to be its dietary fiber. However, further research is needed to elucidate the cause of laxative effect of FBe. These findings suggest that FBe has favorable laxative effects against LP-induced spastic constipation and that oral treatment of SP (5 mg/kg) was more favorable than FBe (300 mg/kg).

## Conclusion

By comparing key factors associated with the laxative effect on the LP-induced spastic constipation in rats, the present work revealed that oral application of 100–300 mg/kg of FBe exhibited promising laxative effects, mediated by increase in gastrointestinal motility. Therefore, FBe may act as a promising functional food ingredient or a laxative agent for the treatment of spastic constipation, with less toxicity observed with the 100 mg/kg dose. The overall laxative effects of FBe 300 mg/kg on LP-induced constipation in rats were milder than those of SP 5 mg/kg; however, there were more favorable increases in intestinal motility in rats treated with FBe 300 and 200 mg/kg than in those treated with SP 5 mg/kg. Furthermore, FBe 100 mg/kg showed similar inhibitory effects on LP-induced decrease in intestinal motility as SP 5 mg/kg.

## Data Availability

All the data used the current study are available with the corresponding author on reasonable request.

## Abbreviations

ANOVA: One-way analysis of variance
Be: Barley extracts
FBe: Triple fermented barley extracts
LP: Loperamide
LSD: Least-significant differences
MW: Mann-Whitney U
SD: Standard deviation
SP: Sodium picosulfate
